# Post Office

**Published:** 1859-10

**Authors:** 


### POST OFFICE.

Nine out of ten of all the complaints against the Post Office Department arise from the stupidity or carelessness of the persons who write or are to receive the letters. Within a short time, three of the newspapers of New York soundly abused the department for loss of monied letters, for days and weeks together, and in each case it was found to be the fault of a thieving message boy.

The merchants and business men of New York seem to have a peculiar fancy for sending boys for their letters and papers, but very few of them are to be trusted : we have frequently seen them drop a paper on the wet floor, and leave it there, because it was a little soiled. We once saw a line of letters strewn from a boy's satchel diagonally across Nassau street from the Post Office, and had he not been called back, he would not have noticed it. These are among the reasons why so many complaints are sent to editors and publishers for missing numbers of newspapers and magazines, and almost always made in an ugly temper, with a peremptory request to send another copy, without offering one time in fifty, to pay for that copy.

During June, 1859, three hundred and twenty-eight letters were sent to the General Post Office, containing evidences of value of over one hundred and seven thousand dollars. Some of these letters had no date or address inside; others had been sent to wrong places; others still, were directed so badly, that it was impossible to decipher the name of either the person or place to which the letter should have gone.

Some letters on being opened contained money, but as the writer failed to name the place from which it was written, there was no means of finding out his residence, and the money was confiscated. The dead letters, that is, those sent to Washington City for the above reasons, or on account of their not being called for in June, contained two hundred and twenty thousand dollars, a large portion of which the owners will never get; to avoid these inconveniencies,—

*First.* Begin your letters by writing in the plainest manner possible, the name of the town, county and state from which the letter is sent.

*Second.* Write your name in full at the close of the letter, without any ornamentation whatever, unless you want to be thought a trifler or a fool, and let every individual letter be perfectly distinct of itself.

*Third.* Let the name of the person to whom the letter is sent be written with equal distinctness, as also the name of the Post office, county and state.

*Fourth.* Put a postage stamp outside, and another inside, if an answer is desired exclusively for your own benefit.

